# Johnson and Johnson COVID-19 Vaccination Triggering Pheochromocytoma Multisystem Crisis

**DOI:** 10.7759/cureus.18196

**Published:** 2021-09-22

**Authors:** Nahel Haji, Sofiah Ali, Emad A Wahashi, Mahrukh Khalid, Kalyana Ramamurthi

**Affiliations:** 1 Internal Medicine, St. Joseph Mercy Oakland Hospital, Pontiac, USA; 2 Nephrology, St. Joseph Mercy Oakland Hospital, Pontiac, USA

**Keywords:** pheochromocytoma multisystem crisis, j&j covid-19 vaccine, rare, triggered, adverse event

## Abstract

Pheochromocytomas are rare tumors that may have variable presentations. The presentation may depend on the type of catecholamine secreted, whether there is a paraneoplastic syndrome or not, or some other factor which may not be well understood. One rare presentation is a pheochromocytoma multisystem crisis. Many of these tumors are asymptomatic and found incidentally, but some can be triggered after being previously dormant. In this case report, we describe the first case of pheochromocytoma multisystem crisis triggered by the Johnson and Johnson (J&J) coronavirus disease 2019 (COVID-19) vaccine. We describe a case of a 63-year-old Caucasian male who presented with intractable nausea, vomiting, dyspnea, watery diarrhea, chills, sweats, and heavy chest pain starting one day status post J&J COVID-19 vaccination. He had no symptoms prior to this and no significant past medical history besides daily marijuana use. During his hospital stay, he had persistent high fevers, respiratory failure, cardiogenic shock, cardiomyopathy, and labile blood pressure measurements. After a retroperitoneal ultrasound, he was found to have a 7 cm mass in the right adrenal gland with elevated chromogranin A, urine vanillylmandelic acid (VMA), and urinary 24-hour metanephrines to confirm the diagnosis of a pheochromocytoma.

## Introduction

Pheochromocytomas are rare catecholamine-secreting tumors arising from the adrenal medulla. Almost all catecholamine-secreting tumors are benign and intra-adrenal, although some can be extra-adrenal and termed catecholamine-secreting paragangliomas [1-5]. Many pheochromocytomas are discovered incidentally on abdominal imaging for unrelated reasons, but symptomatic patients will commonly present with episodic headache, sweating, tachycardia, and paroxysmal hypertension [1,6,7]. The hypersecretion of one or combinations of norepinephrine, epinephrine, and dopamine causes the symptoms of pheochromocytomas [1]. An increase in central sympathetic activity can contribute as well [1]. Patients with tumors that only secrete epinephrine may present with episodic hypotension [8,9]. Pheochromocytomas in asymptomatic patients may be triggered by certain medications as will be discussed later.

A patient with pheochromocytoma multisystem crisis may have hypertension or hypotension, hyperthermia, mental status changes, and other organ dysfunction, similar to our patient's symptoms [10]. In addition, the rapid cyclical fluctuations of hypertension and hypotension along with his cardiomyopathy are also rare manifestations of the condition [11-13]. The mechanism for cyclical hypertension and hypotension is unknown, but fluid repletion and alpha-adrenergic antagonists may help [12,13]. The rarity of this presentation is magnified further by the fact that his condition was triggered by receiving the Johnson and Johnson (J&J) coronavirus disease 2019 (COVID-19) vaccine, an adverse event not previously reported. 

Coronaviruses are single-stranded, positive-sense, enveloped RNA viruses [14]. The COVID-19 virus is a variant of the coronavirus that causes severe respiratory disease and has caused a global pandemic [14]. Vaccines from different companies have been formulated to combat the virus, one of which being the J&J COVID-19 vaccine. This vaccine is a nonreplicating viral vector vaccine that uses genetically modified adenovirus to create a spike protein that the immune system will respond to [15]. Many people have different reactions to these vaccines, but none have been similar to our patient. 

## Case presentation

A 63-year-old Caucasian male with a history only significant for daily marijuana use received the J&J COVID-19 vaccine on May 7, 2021. One day later, he developed intractable nausea, vomiting, dyspnea, watery diarrhea, chills, sweats, and heavy chest pain. In the emergency department (ED), the patient was tachycardic at 120 beats per minute, hypoxic with oxygen 88% on room air, brain natriuretic peptide (BNP) 3421 pg/ml, elevated troponins at 1.29 ng/ml, procalcitonin 25 ng/ml, lactic acid 5.0 mmol/L, white blood cells 19 K/UL, thrombocytopenia at 101 K/UL, and creatinine 1.91 mg/dL. Initial chest X-ray showed bilateral airspace opacities consistent with atypical pneumonia. The patient was intubated because he was in severe respiratory distress with use of accessory muscles and decreased oxygen saturation. Ventilator settings are presented in Table 1. 

**Table 1:** Ventilator settings

Ventilator Setting	Value
Fraction of inspired oxygen (FiO2)	100%
Respiratory rate (RR)	16 breaths per minute
Positive end-expiratory pressure (PEEP)	10 cm H20

Echo showed an ejection fraction (EF) of 25% with global hypokinesis and grade 3 diastolic dysfunction. A right heart catheterization was performed, showing severe pulmonary hypertension. An intra-aortic balloon pump was inserted, but his condition continued to decline, so a left-sided Impella device (Impella Cardiosystems AG, Aachen, Germany) was placed and milrinone and norepinephrine were started.

**Figure 1:**
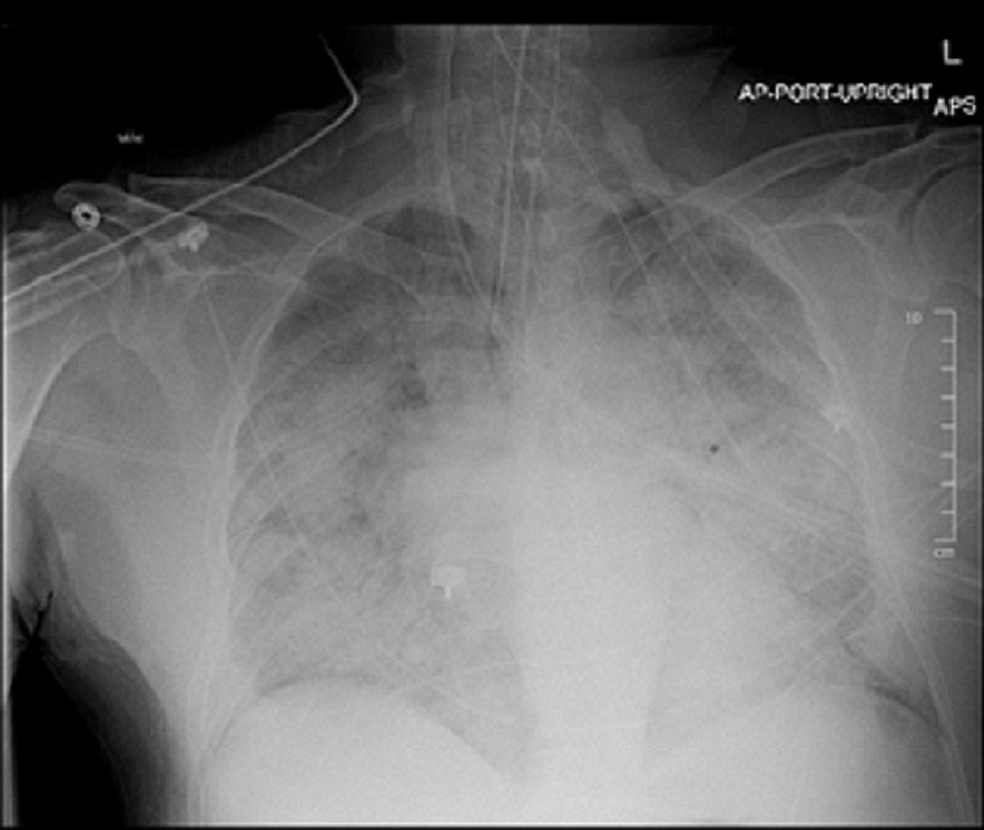
Chest X-ray showing diffuse interstitial and patchy alveolar opacities

The patient was worked up for infectious causes and all were negative including two negative COVID-19 results, viral panel, blood cultures, urine cultures, sputum cultures, and urine legionella. A chest X-ray five days after presenting to the ED is shown below (Figure 1).

Throughout his nine-day hospital stay, he continued to have rapid cyclical fluctuations of hypertension (260s/130s) and hypotension (40s/20s), persistent fevers up to 40.2 °C, and acute respiratory distress syndrome (ARDS)-like picture. His other problems included acute tubular necrosis (ATN) from an acute kidney injury (AKI), hypernatremia, arterial catheter-associated thrombosis in mid left radial artery leading to gangrene of left 1st and 2nd digits, non-ST-elevation myocardial infarction (NSTEMI) with cardiogenic shock (on May 10, 2021, EF was 55% with no diastolic dysfunction), subclinical hyperthyroidism, and electroencephalogram (EEG) showed diffuse slowing, with no epileptiform activity. Echo on May 15, 2021 showed EF of 43% with mild global hypokinesis.

Neuroendocrine crisis was suspected, and the patient was worked up for pheochromocytoma and carcinoid syndrome (Table 2).

**Table 2:** Lab results Values in bold are results above the normal reference range.

Study done	Reference range	Test results
Metanephrine, Plasma	<0.5 nmol/L	31.1
Normetanephrine, Plasma	<0.9 nmol/L	8.9
Urine Vanillylmandelic acid	1.8 - 6.7 mg/day	153.9
24-hour urine total metanephrines	140 - 785 ug/day	54,391
24-hour urine Creatinine	1.0 - 2.0 gm/24h	1.1
24-hour urine metanephrines	52 - 341 ug/day	36,144
24-hour urine normetanephrines	88 - 444 ug/day	18,247
24-hour urine 5-Hydroxyindoleacetic acid	3-10 mg/24h	5.0

**Figure 2:**
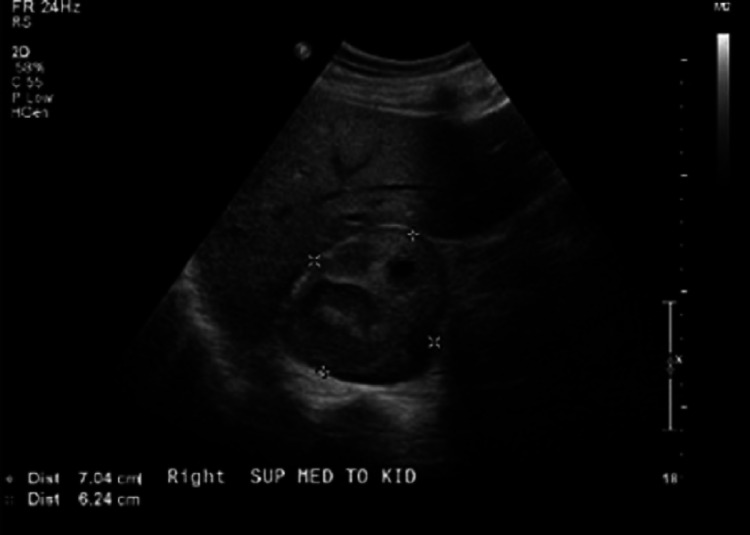
Retroperitoneal ultrasound showing 7.0 x 6.2 x 7 cm heterogenic mass in right adrenal gland

**Figure 3:**
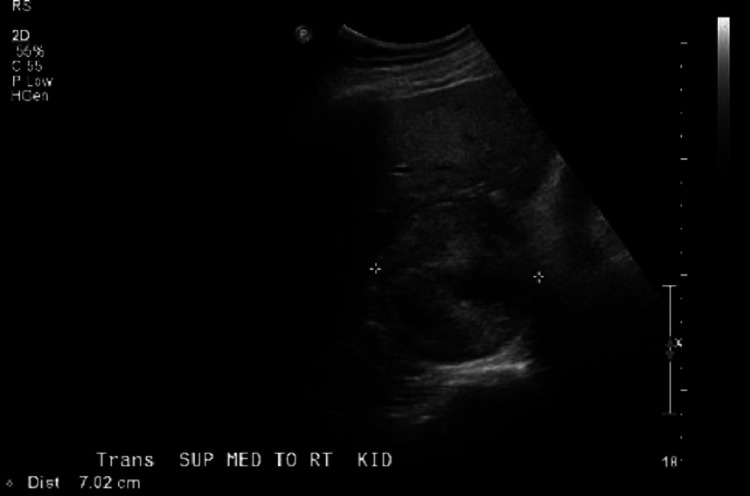
Retroperitoneal ultrasound showing 7.0 x 6.2 x 7 cm heterogenic mass in right adrenal gland

The results of retroperitoneal ultrasound are shown below (Figures 2-3).

The first step in the diagnosis of pheochromocytoma is to discontinue medications that may interfere with metanephrine levels such as norepinephrine. Due to our patient's labile blood pressure measurements, he was on and off of vasopressors (such as norepinephrine) and anti-hypertensive medications (nitroglycerin). The results of significantly elevated 24-hour urine fractionated metanephrines and catecholamines or elevated fractionated plasma metanephrines confirms the diagnosis of pheochromocytoma. Imaging with CT or MRI is done to localize the tumor. Our patient was not stable enough for these imaging tests and thus we had to do an ultrasound. There were no previous imaging studies on record for this patient or a family history that could explain the etiology of his tumor. 

The patient was transferred to the University of Michigan (U of M) hospital for mass resection on May 21, 2021. A CT was performed that day to confirm the ultrasound finding of a 7 cm heterogeneous right adrenal mass. The patient continued to suffer from acute hypoxemic respiratory failure and a tracheostomy was performed on June 07, 2021 due to prolonged intubation. His stay at U of M hospital was also complicated by episodes of non-sustained ventricular tachycardia (NSVT) that responded well to synchronized cardioversion. He was also placed on amiodarone. On June 18, 2021, an open right adrenalectomy was performed and on June 22, 2021, repeat plasma metanephrine was <0.2 nmol/L, and normetanephrine was 0.9 nmol/L. An echo performed on June 22, 2021 revealed a left ventricular EF of 63%, normal overall left ventricular systolic function, normal overall right ventricular systolic function, and no significant valvular dysfunction. Multiple upper and lower extremity deep vein thromboses (DVTs) were found on June 24, 2021. The patient was treated with blood thinners and stabilized for transfer to inpatient rehabilitation on July 02, 2021. The patient was then discharged on July 12, 2021 to stay with his sister. He has continued outpatient therapy and is now able to walk on his own and perform all of his activities of daily living independently. He will follow up with endocrine genetics for the etiology of his pheochromocytoma, cardiology, and physical medicine and rehabilitation. He will continue amiodarone for NSVT and rivaroxaban for the DVTs until the end of September 2021. The patient is doing very well overall. 

## Discussion

Pheochromocytomas are rare tumors with an annual incidence of 0.8 per 100,000 person-years [16]. They are catecholamine-secreting tumors arising from chromaffin cells of the adrenal medulla. Almost all catecholamine-secreting tumors are intra-adrenal, while about 10%-15% are extra-adrenal, typically along the sympathetic ganglia in the posterior mediastinum [1,2]. Approximately 90% of pheochromocytomas are benign, with the remaining minority being metastatic [1,3-5].

While about 60% of pheochromocytomas are discovered incidentally on abdominal imaging for unrelated reasons, common symptoms for symptomatic patients include episodic headache, sweating, tachycardia, and paroxysmal hypertension [7]. Our patient had no such symptoms prior to his presentation. 

Rarely do patients present with pheochromocytoma multisystem crisis and rapid cyclical fluctuations of hypertension and hypotension. Our patient’s development of cardiomyopathy with global hypokinesis along with the fact that his condition was triggered by receiving the J&J COVID-19 vaccine add further to the uniqueness of this case. Given the large size of his tumor, it is unlikely that the tumor appeared suddenly and caused a pheochromocytoma crisis in our patient. The tumor had most likely been growing for months or even years while the patient remained asymptomatic until something triggered its activity. The J&J COVID-19 vaccine is the only logical trigger as it was given one day before his symptoms began and there were no other inciting factors including medications, drugs, or infection. This sequence of events makes logical sense because vaccines trigger an immune response similar to how an infection would and this likely triggered the activity of the pheochromocytoma. How exactly this occurs remains to be discovered and further studies will be needed to describe this interplay. 

A review of the literature revealed that the adverse events after the J&J vaccine included 14 cerebral venous sinus thrombosis (CVST) cases, three non-CVST thrombocytopenia syndrome, four cases of confirmed anaphylaxis, and 88 reported deaths [17]. Many cases reported no details as to the cause of death but 23 were from cardiac arrest or cardiovascular disease, eight from COVID-19 disease, and five from cerebrovascular disease [17]. There were no reports of anything similar to our patient’s case. There is a report of a pheochromocytoma being triggered by high-dose exogenous steroids and another that describes cases of hypertensive crisis in patients with pheochromocytomas being triggered by certain drugs including rocuronium, pancuronium, and atracurium [18,19].

Upon further review of the literature, there have been 47 cases of pheochromocytoma presenting clinically as shock from 1966-2003 [20]. Certain drugs such as marijuana were used in 30% of patients prior to the onset of shock and our patient used marijuana daily [20]. While this is interesting to note, our patient used marijuana daily, and for the months or years that he had the tumor, it never caused any symptoms. Thus, it is improbable that it triggered a pheochromocytoma crisis on one particular day when it had not done so in the past. The mechanism of shock caused by pheochromocytomas is not fully understood but is thought to be due to hypovolemia from increased capillary permeability or from myocardial injury leading to decreased cardiac output [20]. The possibility of pheochromocytomas secreting interleukin-6 (IL-6) in a paraneoplastic fashion causing an acute inflammatory syndrome has also been reported [21,22]. Levels of IL-6 were not measured in our patient; thus, it is not clear whether this was a contributing factor or not. Our patient presented similarly to those with shock, or an acute inflammatory syndrome as described above, but his symptoms most closely resemble a pheochromocytoma multisystem crisis.

## Conclusions

Pheochromocytomas are rare and benign tumors. Most are asymptomatic and rarely present with multisystem organ dysfunction and labile blood pressures when symptoms are present. A pheochromocytoma crisis in previously asymptomatic patients may be triggered by certain medications (such as rocuronium and corticosteroids) or as in our patient, the J&J COVID-19 vaccine. Further studies will be needed to investigate how exactly these medications and the J&J COVID-19 vaccine cause a pheochromocytoma crisis. Diagnosis can be confirmed with significant elevations in biochemical tests and abdominal imaging can localize the tumor for eventual resection. Some pheochromocytomas do not present with classic symptoms of paroxysmal hypertension, episodic headache, sweating, and tachycardia; thus, it is prudent to remain suspicious when patients present with sudden symptoms of multiorgan dysfunction, labile blood pressures, and hyperthermia suggesting the triggering of a previously dormant pheochromocytoma.
